# The site of the bite: *Leishmania* interaction with macrophages, neutrophils and the extracellular matrix in the dermis

**DOI:** 10.1186/s13071-016-1540-3

**Published:** 2016-05-04

**Authors:** Juliana Perrone de Menezes, Elvira M. Saraiva, Bruno da Rocha-Azevedo

**Affiliations:** Gonçalo Muniz Research Center, Oswaldo Cruz Foundation (FIOCRUZ), Salvador, Brazil; Departamento de Imunologia, Instituto de Microbiologia Paulo de Góes, Universidade Federal do Rio de Janeiro (UFRJ), Rio de Janeiro, Brazil; Programa de Terapia Celular e Bioengenharia, Instituto de Biofísica Carlos Chagas Filho, Universidade Federal do Rio de Janeiro (UFRJ), Rio de Janeiro, Brazil; Present Address: Department of Biophysics, The University of Texas Southwestern Medical Center, 5323 Harry Hines Boulevard, Dallas, TX 75390 USA

**Keywords:** *Leishmania*, Extracellular matrix, Macrophages, Neutrophils

## Abstract

*Leishmania* spp., the causative agents of leishmaniasis, are intracellular parasites, transmitted to humans via the bite of their sand fly vectors. Once inoculated, the promastigotes are exposed to the dermis, which is composed of extracellular matrix (ECM), growth factors and its resident cells. Promastigote forms are phagocytosed by macrophages recruited to the site of the sand fly bite, either directly or after interaction with neutrophils. Since *Leishmania* is an intracellular parasite, its interaction with the host ECM has been neglected as well as the immediate steps after the sand fly bite. However, promastigotes must overcome the obstacles presented by the dermis ECM in order to establish the infection. Thus, the study of the interaction between *Leishmania* promastigotes and ECM components as well as the earliest stages of infection are important steps to understand the establishment of the disease, and could contribute in the future to new drug developments towards leishmaniasis.

## Background

Leishmaniasis is one of the neglected tropical diseases, with 350 million people in 98 countries worldwide at risk of developing one of the many forms of the disease. This disease is caused by different species of the genus *Leishmania* - heteroxenous flagellated protozoa that mainly infect macrophages of mammalian tissues. Parasite entry into the dermis of the mammalian host occurs by innoculation during the bite of the sand fly vector during a blood meal [1]. Promastigotes are inoculated in a pool of blood where they interact with leukocytes [2, 3]. Thus, most host-*Leishmania* interaction studies have focused on the interaction between *Leishmania* promastigotes and their cellular targets (dermal dendritic cells, neutrophils, and macrophages) [4]. However, at the earliest step of infection after the sand fly bite - promastigotes could be deposited into the extracellular matrix (ECM) of the dermis and the blood. In this event, promastigotes interact with extracellular matrix and basement membrane proteins [5], before infecting their cellular targets [3]. In this review, we focus on some of the advances in the cell biology of the early stages of the interaction between *Leishmania,* the dermis microenvironment, composed of its ECM and immune cells such as neutrophils and macrophages.

## Interaction between *Leishmania* and host extracellular matrix

The ECM is a complex, tissue specific network of biomolecules that gives shape and physical attributes to tissues and also acts as an environmental cue to the cells surrounding its structure [6, 7]. The composition and the shape of the ECM leads to distinct cell behavior, such as survival, differentiation, and proliferation as well as cell migration and tissue invasion [8, 9]. Collagen I is the major ECM component of the skin, and fibroblasts are the cells responsible for its synthesis and organization [10]. The other important component of the skin ECM is the basement membrane, composed of laminin and collagen IV, which separates the dermis from the epithelial layer [11]. During injury, skin structure is damaged, leading to the activation of wound healing, which is a multistep process that requires the action of fibroblasts, immune cells and a myriad of growth factors, cytokines and matrix components [11, 12].

During a sand fly blood meal significant damage occurs to the structure of skin, leading to rupture of the dermis and its capillaries, creating a blood “pool” containing ECM components from tissue and blood, and different cells as well [13, 14]. This wound milieu at the dermis attracts macrophages, which are the main host cell type of *Leishmania* [15] and neutrophils as well [16]. Thus, a synchronized action mediated by promastigotes towards immune cells and ECM will be required for the establishment of the infection. Parasites will need to migrate in a very complex extracellular environment before being internalized by neutrophils or macrophages. Thus, the direct study of the interaction between *Leishmania* promastigotes and ECM components is an important step to understand the establishment of the infection.

Experimental leishmaniasis models have shown that collagen I, the major ECM component of the skin [17], is the predominant ECM component found in early stage lesions [18]. In vitro studies demonstrated the ability of promastigotes to attach to and move through collagen I scaffolds [19]. Attachment to collagen I occurred in a dose-dependent manner, indicating the presence of a parasite surface receptor [20]. Furthermore, parasite invasion on collagen I 3D scaffolds led to collagen remodeling (about 20 % degradation), possibly mediated by metallo- and cysteine proteinases. Physical traits of the matrix, such as stiffness, decreased promastigote migration [19]. These observations may indicate that promastigotes need to secrete proteases to breakdown rigid collagen scaffolds to facilitate migration in the host before being internalized by a host cell. Interestingly, a collagen “shift” occurs during experimental infection with *Leishmania*: collagen I observed in early stages is substituted by collagen III during chronic phase of the infection [21]. It has been reported that decreased amounts of collagen III result in scar formation due to myofibroblast differentiation [22]. In contrast, a higher content of collagen III during leishmaniasis can induce the presence of a softer skin matrix, by which parasites would have an easier path for migration and tissue invasion. Promastigotes also bind to collagen VI, which is normally secreted by macrophages [23].

Fibronectin (FN), an adhesive glycoprotein found in the blood and connective tissues [24], is possibly found in sand fly mosquito bite lesions. An increase in FN expression in *Leishmania*-infected tissue has been observed in murine models [18]. Surface proteins from both promastigotes and amastigotes bind to FN, facilitating monocyte uptake, since these cells recognize FN [25]. Gp63 - a surface metalloproteinase - and cysteine proteases are able to degrade FN. These FN fragments inhibit macrophage formation of reactive oxygen species, which are important in clearance of the infection and also facilitate parasite and macrophage migration [5, 26], helped also by FN shedding [27]. Gp63, also is a FN-like molecule [28], which is recognized by macrophage integrin receptors contributing to parasite internalization [28]. A SYRD tetrapeptide from gp63 mimics the classic integrin recognition binding site RGDS [29], confirming its identity of FN-like molecule for this enzyme.

Another major group of ECM components present in the blood is the plasminogen/plasmin/fibrin system. Critical for coagulation, the blood clot also provides a provisional matrix by which neutrophils and macrophages migrate to during wound healing processes [30]. Promastigotes will need to escape from this fibrin structure, and at the same time allow target cells to get closer. Likewise, anti-coagulants found in the sand fly saliva are very important not only inhibiting blood coagulation but also counteracting promastigotes procoagulant activity [31]. The reason for this controlled balance of anti- and pro- coagulation molecules is still unknown, but it possibly gives to the dermal clot milieu the proper mechano-biochemical trigger for parasite cell invasion. It is known that *Leishmania* can bind to plasmin and plasminogen [32]. Enolase, a ubiquitous metabolic enzyme binds to plasminogen as a unique example of a multifunctional protein [33]. Promastigotes appear to secrete enolase in exocytic vesicles, which can help in immune evasion [34], since these vesicles have been implicated in parasite-macrophage communication. Furthermore, plasminogen-associated vesicles can trap macrophages, potentially allowing parasites to move further into the dermis.

The basement membrane (BM) is a fundamental structure in the skin. It separates the epithelium from the dermis and also surrounds blood capillaries. Sand fly bites locally disarrange BM structure, and promastigotes are exposed to its components: laminin, collagen IV, nidogen, and perlecan [14, 35]. Laminin is a family of glycoproteins that together with collagen IV form the general BM scaffold [36]. *Leishmania* promastigotes possess a cell surface 67 kDa laminin-binding protein (LBP) [37], which is stimulated by zinc, suggesting a downstream signaling pathway [38]. This LBP appears to be important in *Leishmania* pathogenesis, since administration of anti-LBP antibodies reduce infectivity of *L. donovani* in mouse models [39]. Matrigel, a reconstituted basement membrane scaffold, has been used as substrate to study promastigote migration [5]. The role of the metallo-proteinase gp63 on migration through and degradation of matrigel, was shown to be specifically related to collagen IV degradation, but not laminin [5, 40].

Glycosaminoglycans are also components of mammalian ECM, usually associated with proteins, forming proteoglycans. They are present in the skin and can influence a variety of events during tissue repair, such as avoiding water lost, preventing tissue compression and regulating cell migration and survival [41]. *Leishmania* promastigotes bind to heparan sulfate, heparin and hyaluran [23], present at the dermal epithelial junction and the dermis. A heparin-binding protein, which mediates parasite – proteoglycan binding has been described and implicated in parasite virulence and host cell invasion, since *Leishmania* parasites were unable to invade cells without heparin on the surface. Also, parasite pre-treatment with heparin blocked binding to macrophages [42, 43].

The complexity of the ECM at the site of the sand fly bite increases when we take in consideration all the previously mentioned components in a dynamic setting containing cells. For instance, fibrosis formation mediated by fibroblasts can be a limiting situation for parasite propagation in the dermis. Thus, it is of interest to study cells not infected with *Leishmania* but present in the dermis microenvironment. Despite reports on *Leishmania* infection of fibroblasts [44–46], possibly the major issue is the remodeling of the collagen matrix by fibroblasts, due to growth factor stimulation coming from macrophages [12]. Complex macrophage -3D collagen I interaction system has been described, showing promastigotes migrating on collagen I scaffolds before being internalized by macrophages [19]. Furthermore, parasite migration is faster when macrophages are present [19], possibly indicating that secretion of cytokines can be chemotactic for *Leishmania* in complex in vitro invasion models.

The examples commented in this session are a reminder of how important the extracellular milieu can be critical for the pathogenesis of an infection caused by an intracellular pathogen such as *Leishmania*. Most of *Leishmania*/host cell studies use macrophages and neutrophils in suspended or 2D cultures, which does not reflect the complexity of the dermis during early stage leishmaniasis.

## Interaction between *Leishmania* and neutrophils

Neutrophils are the first cells to migrate to infected sites, where they release antimicrobial mediators and phagocytose microorganisms, and kill infectious agents by generating a potent oxidative burst and releasing toxic mediators into the parasitophorous vacuole. The first studies on *Leishmania*-neutrophil interaction were published in 1981 [47–49], reporting the ability of human neutrophils to phagocytose and kill *L. donovani* promastigotes and amastigotes. Following these first reports, several aspects of the neutrophil-*Leishmania* interaction were studied, which were recently reviewed [50–54].

Here, we will address a recently described property of neutrophils, which is the ability to release their nuclear DNA associated with granular and cytoplasmic proteins to the extracellular milieu [55]. These structures, known as neutrophil extracellular traps (NETs), are released as a scaffold that ensnares and kills microorganisms. The NETs release culminates with a cell death process named NETosis, which is different from apoptosis and necrosis [56]. More recently, another NET release mechanism was described, in which nuclear budding vesicles carrying chromatin are extruded from neutrophils, releasing their contents in the extracellular milieu, where chromatin traps and kills pathogens [57]. This mechanism occurs preserving neutrophil viability and, thus, it was named “vital or early NETosis” to differentiate it from the classic NETosis associated with neutrophil death [58]. Therefore, it is important to address the role of NETosis in leishmaniasis, since the encounter of *Leishmania* and neutrophils is an early event when promastigotes are deposited in a pool of blood formed at the site of the sand fly bite, where neutrophils are abundant [59–65].

Several microorganisms induce NETosis [66] and, among parasites, *L. amazonensis, L. major* and *L. infantum* promastigotes [67] elicit the classical NETosis after in vitro interaction with human neutrophils. It is known that the majority of microorganisms induce the classical NETosis, which is dependent on reactive oxygen species (ROS) generated by NADPH oxidase, and of chromatin decondensation mediated by peptidylarginine deiminase 4 (PAD4), elastase and myeloperoxidase [56, 68–70]. It is only known that early NETosis, on the other hand, occurs quickly (5–15 min) after inducer-neutrophil interaction and that it is independent of ROS generation [57, 58]. Recently, we demonstrated that *Leishmania* promastigotes triggered both NETosis mechanisms, the classic one occurring with redox imbalance, PAD4 and elastase participation, and the early NETosis occurring in an elastase-dependent and ROS and PAD4-independent manner [71].

We have shown that *L. amazonensis* promastigotes were caught and killed by NETs in a histone-mediated mechanism [67]. The parasite death was evaluated by cell morphology in scanning electron microscopy and by quantifying promastigote survival in cultures with neutrophils in which DNase was added to disrupt NET-DNA mediated death [67]. Although the mechanism of histone-mediated cell death is still unknown, histone toxicity has been demonstrated for to promastigotes of *L. mexicana, L. braziliensis, L. major* and *L. amazonensis,* but histone resistance has been reported for amastigotes of *L. mexicana* and *L. amazonensis* [72]. Interestingly, *L. donovani* promastigotes induced NET extrusion by human neutrophils, although they were resistant to NET mediated killing, protected by the lipophosphoglycan (LPG) expressed on the promastigote cell surface, since LPG-knockout parasites were susceptible to the NET-killing mechanism [73]. In contrast to *L. donovani* LPG, purified *L. amazonensis* LPG triggered NETosis [67], a difference that could be due to the inter- and intra-specific polymorphisms found in this molecule of different *Leishmania* species [74, 75].

More recently, it has been demonstrated that *L. mexicana* induced NETosis in mice neutrophils in vitro, and NETs were observed in the ear of mice inoculated with this same parasite. Interestingly, these NETs did not kill *L. mexicana* promastigotes [65].

Properties already assigned to NETs include trapping to avoid pathogen spreading, phagocytosis assistance through its trapping activity, and killing of microorganisms. Microbes have also evolved different strategies to escape NETs toxic mechanisms. Accordingly, a conserved bacterial tool to escape NET toxicity is the expression of nucleases, which efficiently degrade NET-DNA scaffolds, neutralizing their toxic effects and allowing bacterial spread throughout the body [76–84]. Interestingly, *Leishmania* promastigotes express 3’-nucleotidase/nuclease, a class I nuclease member, which cleaves NET-DNA, allowing parasites to escape NET-trapping and -killing [85]. Furthermore, the *Lutzomyia longipalpis* sand fly saliva inoculated into the host skin together with *Leishmania* promastigotes, contains the powerful endonuclease *Lutzomyia* NET destroying protein (Lundep), that degrades NET-DNA meshes, allowing parasites to escape NET-toxic activity and exacerbates *Leishmania* infection [86]. The evolutionary conservation of NET induction ability into different *Leishmania* spp., points to the possibility that NETosis occurs during parasite transmission to the vertebrate host; however, NETs toxicity might be counteracted by the activity of promastigote nucleases, as well as by the presence of endonuclease in the vector’s saliva. Importantly, not only promastigotes induce NETosis, since amastigote forms also trigger NETs in vitro [62], and amastigote nests were found associated with NETs in lesions of human American tegumentary leishmaniasis [87]. The study of the *Leishmania* ability to induce NET release could advance the understanding of the early aspects of the innate immunity to this protozoan and of the pathogenesis of *Leishmania* infection as well.

## Interaction between *Leishmania* and macrophages

*Leishmania* promastigotes are rapidly phagocytized by neutrophils and macrophages after being inoculated by the sand fly vector. Although *Leishmania* is mainly found in neutrophils during the first hours of infection, the parasites do not differentiate into amastigotes inside these cells, but in macrophages. Thus, macrophages are important for the establishment of infection and persistence of the parasite inside the host [64, 88]. In addition, it has been previously reported that upon phagocytosis of *Leishmania major*, mononuclear phagocytes harboring live parasites migrate from the skin to the draining lymph node of the host [89–91]. Macrophage migration is dependent on the interaction of these cells with the ECM [92], although the mechanisms involved in this process during *Leishmania* infection remains unknown. The capacity of these cells to home to the skin, to mucosae, or to internal organs may also be modulated by the parasite [93] and can play an important role in the dissemination of the disease.

Studies using *L. mexicana* and *L. infantum* have shown that molecules like proteophosphoglycans secreted by these parasites in the sand fly’s midgut and inoculated into the host during blood meal are powerful stimulators of macrophage recruitment [94, 95]. During the initial recognition events, different species of *Leishmania* rely on a range of macrophage receptors, including complement receptors (CRs), mannose receptors (MR), fibronectin receptors and Fcγ receptors (FcγRs), which may later impact the course of infection [28, 96–101]. Although reports in the literature have claimed that these routes of parasite entry are redundant, the ligation of specific receptors elicits different downstream functions in the macrophage [28, 96–101]. It was shown that avirulent promastigotes in logarithmic growth enter parasitophorous vacuoles lined with CR3 and MR, whereas PVs surrounding density-purified metacyclics contained only CR3 [102]. CR3, but not MR, clusters in cholesterol- and caveolin-containing microdomains, which were previously characterized as entry routes that direct *L. infantum* promastigotes into a pathway that leads to a 24- to 48-h delay in lysosomal fusion and allows better replication of parasites, leading to intracellular survival [103].

Once the parasite is recognized by the macrophage, focal exocytosis of host cell membrane originating from endosomes, lysosomes and the endoplasmic reticulum contribute to the formation of the promastigote-containing phagosomes [104–106]. Such supply of membrane from various intracellular compartments may contribute to the formation and the composition of the nascent parasitophorous vacuole. Other factors during the initial moments of the infection have an important role in determining the establishment of the disease, such as the sand fly saliva. *Leishmania* parasites as well as sand fly saliva have been associated with suppression of the initial proinflammatory immune response, promoting parasite survival [107–111]. Co-injection of saliva or its components together with *Leishmania* were shown to exacerbate cutaneous leishmaniasis, producing larger lesions and a higher parasite burden. This enhancement in *Leishmania* lesions by saliva was attributed to the immunomodulatory properties of the salivary proteins, which act in the initial moments of the infection, promoting downregulation of macrophage and dendritic cell functions and the production of anti-inflammatory cytokines that favor parasite survival and establishment [112–116]. It has been shown that the increase in infectivity was associated with the ability of the saliva to selectively inhibit pathogen recognition, nitric oxide (NO) and hydrogen peroxide production thus, inhibiting the ability of macrophages to kill the parasites [117, 118]. Furthermore, it was also shown that *Leishmania* vector saliva inhibits the production of protective type 1 cytokines such as IL-12 and IFN-γ, while enhancing the production of IL-10, IL-4, IL-6 and prostaglandin E (PGE)_2_, all of which enhance parasite survival [108, 119, 120].

After the inoculation and initial infection, *Leishmania* parasites may remain at the inoculation site or disseminate in the host tissues. Although the mechanisms that control *Leishmania* dissemination through different host tissues are poorly understood, the initial events that occur at the site of infection have an important role in this process. Evidence suggests that *Leishmania* infection and the parasite burden modulate the adhesion and migratory capability of mononuclear phagocytes [121, 122]. Carvalhal and coworkers [121] demonstrated that infection with different *Leishmania* species impairs the adherence of monocytes and macrophages to connective tissue. Such impairment in leukocyte adhesion is due to interference with integrin function, as the authors demonstrated a regulation in cell surface β1-integrin activity in infected macrophages [123]. Furthermore, infection with *Leishmania* downregulates the expression of the genes encoding the chemokine receptors CCR4 and CCR5 in murine dendritic cells. The impairment of chemokine production can be related to reduced migration of phagocytes from the parasite inoculation site and could interfere with the development of a systemic/adaptive response [121, 122]. In addition, it has been shown recently that leukocyte spreading over a fibronectin-coated surface is abrogated in *Leishmania*-infected cells [124]. These changes in the initial moments of the infection and in phagocyte function may be important for parasite dissemination and distribution of lesions in leishmaniasis.

## Conclusions

Although long viewed only as a supportive structure, the ECM is an essential part of the cell’s milieu that regulates almost all cellular behavior [125], including inflammatory signaling [126]. In leishmaniasis, once *Leishmania* parasites are inoculated in the host’s skin, they will need to migrate in a very complex extracellular environment before being internalized by neutrophils and/or macrophages, which are also responding to a complex wound tissue microenvironment.

Challenges remain in understanding leishmanial biology and how this parasite interacts with the host tissue. However, in the last few years, it seems clear that the initial interaction between *Leishmania* parasites and the host extracellular matrix and immune cells, such as neutrophils and macrophages, has an important role on the determination of *Leishmania* infection outcome. Promastigote ECM migration, fibroblast wound response, macrophage migration, and NET formation can be critical emergent topics for the full understanding of *Leishmania* pathogenesis.
